# Randomized Controlled Study of a Remote Flipped Classroom Neuro-otology Curriculum

**DOI:** 10.3389/fneur.2017.00349

**Published:** 2017-07-24

**Authors:** Frederick Robert Carrick, Mahera Abdulrahman, Ahmed Hankir, Maksim Zayaruzny, Kinda Najem, Palita Lungchukiet, Roger A. Edwards

**Affiliations:** ^1^Neurology, Bedfordshire Centre for Mental Health Research, in association with University of Cambridge, Cambridge, United Kingdom; ^2^Neurology, Carrick Institute, Cape Canaveral, FL, United States; ^3^Medical Education, Harvard Macy and MGH Institutes, Boston, MA, United States; ^4^Department of Medical Education, Dubai Health Authority, Dubai, United Arab Emirates; ^5^Psychiatry, Bedfordshire Centre for Mental Health Research, in association with University of Cambridge, Cambridge, United Kingdom; ^6^Psychiatry, Carrick Institute, Cape Canaveral, FL, United States; ^7^Anesthesiology, University of Massachusetts Medical School, Worcester, MA, United States; ^8^Neuro-Ophthalmology, University of Montreal Medical School, Montreal, QC, Canada; ^9^Emergency Department, Bumrungrad International Hospital, Bangkok, Thailand; ^10^Health Professions Education, MGH Institute of Health Professions, Boston, MA, United States

**Keywords:** neuro-otology, medical education, online learning, classroom learning, flipped classroom

## Abstract

**Context:**

Medical Education can be delivered in the traditional classroom or *via* novel technology including an online classroom.

**Objective:**

To test the hypothesis that learning in an online classroom would result in similar outcomes as learning in the traditional classroom when using a flipped classroom pedagogy.

**Design:**

Randomized controlled trial. A total of 274 subjects enrolled in a Neuro-otology training program for non-Neuro-otologists of 25 h held over a 3-day period. Subjects were randomized into a “control” group attending a traditional classroom and a “trial” group of equal numbers participating in an online synchronous Internet streaming classroom using the Adobe Connect e-learning platform.

**Interventions:**

Subjects were randomized into a “control” group attending a traditional classroom and a “treatment” group of equal numbers participating in an online synchronous Internet streaming classroom.

**Main outcome measures:**

Pre- and post-multiple choice examinations of VOR, Movement, Head Turns, Head Tremor, Neurodegeneration, Inferior Olivary Complex, Collateral Projections, Eye Movement Training, Visual Saccades, Head Saccades, Visual Impairment, Walking Speed, Neuroprotection, Autophagy, Hyperkinetic Movement, Eye and Head Stability, Oscilllatory Head Movements, Gaze Stability, Leaky Neural Integrator, Cervical Dystonia, INC and Head Tilts, Visual Pursuits, Optokinetic Stimulation, and Vestibular Rehabilitation.

**Methods:**

All candidates took a pretest examination of the subject material. The 2–9 h and 1–8 h sessions over three consecutive days were given live in the classroom and synchronously in the online classroom using the Adobe Connect e-learning platform. Subjects randomized to the online classroom attended the lectures in a location of their choice and viewed the sessions live on the Internet. A posttest examination was given to all candidates after completion of the course. Two sample unpaired *t* tests with equal variances were calculated for all pretests and posttests for all groups including gender differences.

**Results:**

All 274 subjects demonstrated statistically significant learning by comparison of their pre- and posttest scores. There were no statistically significant differences in the test scores between the two groups of 137 subjects each (0.8%, 95% CI 85.45917–86.67952; *P* = 0.9195). A total of 101 males in the traditional classroom arm had statistically significant lower scores than 72 females (0.8%, 95% CI 84.65716–86.53096; *P* = 0.0377) but not in the online arm (0.8%, 95% CI 85.46172–87.23135; *P* = 0.2176) with a moderate effect size (Cohen’s *d* = −0.407).

**Conclusion:**

The use of a synchronous online classroom in neuro-otology clinical training has demonstrated similar outcomes to the traditional classroom. The online classroom is a low cost and effective complement to medical specialty training in Neuro-Otology. The significant difference in outcomes between males and females who attended the traditional classroom suggests that women may do better than males in this learning environment, although the effect size is moderate.

**Clinical Trial Registration:**

Clinicaltrials.gov, identifier NCT03079349.

## Introduction

The increasing availability of online learning has increased the ability of medical students, residents, fellows, and practicing physicians to learn at a distance. Health-care providers have become increasingly dependent upon technological methodology associated with clinical practice. We have identified a need to quantify and qualify the integrity of medical education programs that utilize current technology and a further need to choose the best technology for the educational task. The online learning environment should be contemporary in order to adequately present medical education and to contribute clarity to comparisons of learning environment. We needed to develop a methodology of instruction that is evidence based and current in order to test our hypothesis that there is no difference in learning associated with a traditional classroom and a synchronous online classroom experience.

There are large numbers of educational platforms commercially available, and we had a need to review and understand screencasting best practices in order to develop a screencasting platform. Screencasting, the capture of computer screen and audio content for future dissemination, allows educators to guide viewers through the content presented. Viewers are thus able to engage the material at their convenience. In 2014, Andrew Youngkin described the benefits of screencasting in health professional education (1). Such benefits included the enhancement of demonstrations, facilitation of collaborative activities, encouragement of communication, and the evaluation of learning/understanding. Youngkin discussed the use of the “flipped classroom” and suggested opportunities for further exploration (2). Youngkin noted the use of the “flipped classroom” to improve efficiency in curriculum delivery and foster in-depth class discussion and collaborative problem solving.

We have decided to use a flipped classroom in the development of our coursework. It was our hope that we would also be able to improve the efficiency in our curriculum delivery both in the live class and in a live streaming class. Ashley Woodruff’s group implemented and assessed the effectiveness of a hybrid learning model using screencasting with embedded assessments in pathophysiology and therapeutics modules (3). Their results showed the hybrid model offered a novel method to afford students active learning opportunities to progress to higher cognitive domains of learning. The implementation of a hybrid model was attractive to us as the subject material we teach in neuro-otology is complex and educational models that will facilitate higher cognitive domains of learning were ideal.

Amanda Lackey and her group suggested screencasting is an opportune time for radiologists to focus on personal productivity and noted that the ever increasing reliance on computers and the Internet has significantly changed the way we work (4). They discussed the use of screencasting and other tools that help improve collaboration and personal productivity, maximize e-learning, and protect valuable digital data. The concept of being able to share the computer screen outside of the live classroom experience was a major part of our project. We desired to deliver a robust educational experience to physicians that might not be able to attend the live classroom. An understanding of screen casting in a medical educational model was certainly obtainable.

Louw et al. described local and global factors’ impact on the need for and provision of the limited transfusion medicine education in South Africa. He suggested the inclusion of screencasting and online learning into teaching programs (5). Our learners are dispersed throughout the world and have told us that they desired an online learning experience. Roshan Razik and colleagues studied the preferences and attitudes of ophthalmologists toward screencasting (6). They found practicing ophthalmologists and residents were interested in screencasting. However, rural ophthalmologists preferred live lectures compared to urban colleagues. The social atmosphere of an onsite learning environment appeared to benefit those ophthalmologists that lived and worked in an area without daily professional interplay. These differences highlight the importance of knowing the audience when using screencasting.

We desired to find out if there was a preference of learning between streaming through screencasting and live lectures. We also wanted to find out if the outcomes would be similar so that we might embrace an evidence-based design of an online learning program. With existing technology, library users have many options for accessing information. Stephanie Kerns presented how screencasting and other technologies increased flexibility, enhancing learning in many educational settings (7). This was helpful to us as we had to be able to choose a platform that would enable us to deliver high quality medical education to an international audience. In order to do this, we needed to understand our audience, their needs, and their learning characteristics. We also knew that our planning of the educational experience would be dependent upon our ability to share it well. We realized that any educational program should be evidence based and contemporary both from a pedagogical and technical perspective. We needed to develop a curriculum that was outcome based with specific goals defined before the curriculum was designed. We also wanted to ensure that our curriculum was designed specific to an outcome that would increase the application of neuro-otology skills.

The flipped classroom is an educational model with the potential to improve the learning environment where students gain exposure to new material outside class and then use class time to assimilate the knowledge through problem-solving exercises or discussion (7). Thus the typical lecture and homework elements of a course are reversed or flipped. Thus, the flipped classroom is a learner centered approach in which the learner is responsible to attend the class with a basic understanding of the subject to fully participate and engage in discussions (8). Flipping a classroom has a number of potential benefits, for example increased educator-student interaction, but must be planned and implemented carefully to support effective learning (9). Sarah McLean and colleagues from Western University in London, ON, Canada, suggested that there is the potential for greater educational gains from the flipped classroom than the modest improvements in grades previously demonstrated in the literature (10). They also found that implementation of the flipped classroom resulted in students reporting that they developed independent learning strategies, spent more time on task, and engaged in deep and active learning.

Eunicia Tan and colleagues evaluated the relative acceptability of the flipped classroom approach compared with traditional didactics for in-house teaching in emergency medicine and concluded that the flipped classroom shows promise as an acceptable approach to in-house emergency medicine teaching (11). Helen Morgan and her group from the University of Michigan found that their implementation of the flipped classroom curriculum for gynecologic oncology topics successfully demonstrated a promising platform for using technology to make better use of their students’ time and for increasing their satisfaction with the necessary didactic learning of the clerkship (12). Brent Orndorff and colleagues at Indiana University School of Medicine identified that distance education has been a reality for many years in the form of combining traditional classroom instruction with web-based educational technologies (13). They noted that limitations remain in established technologies that restrict the types of courses offered through this medium. They initiated a program to overcome these limitations and allow for a more interactive learning experience. They were able to successfully combine Adobe Connect software with Polycom videoconferencing. Their experimentation with the integration of technologies has allowed them to enhance the level of interactivity of student learning promoting the remote delivery of the medical school curriculum.

Other investigators have described the use of Adobe Connect software along with algorithm software to provide the necessary audio visual communication platform for telementoring a complex medical procedure to novice providers located at a distant site (14). The teaching efficacy of multipoint video teleconferencing is perceived by medical residents to be more effective when complemented by application-sharing software such as Adobe Acrobat Connect (15). The development of an online learning program must utilize appropriate technology and identify problems in learning that maybe associated with distance learning. Manwa Ng and colleagues have identified problem-based learning (PBL) as an effective pedagogical tool for promoting student competencies in self-directed and collaborative learning, critical thinking, self-reflection as well as adapting to novel situations (16). They identified that the need for face-to-face interactions at the same place and time severely limits the potential of traditional PBL. The requirements of space and for meeting at a specific location at the same time can create timetabling difficulties. Ng’s team designed and implemented an online PBL environment using Adobe Connect for undergraduate speech/language pathology students, and assessing the associated pedagogical effectiveness. They found that all students enjoyed online PBL, without any perceived negative effects on learning and statistical analysis indicated no significant difference in assignment grades between the online and traditional PBL groups, indicating that online PBL learning appears to be similarly effective as traditional face-to-face PBL learning.

Neuro-otology is a specialty discipline that involves the skill and training to evaluate parts of the brain and nervous system that are associated with hearing and balance in defining the cause of a patient’s symptoms. A high level of understanding of the brain and vestibular system is necessary to develop meaningful treatment paradigms for dizzy patients and for those with disorders of balance, station, and gait. Our neuro-otology training program for non-neuro-otologists prepares physicians to master complex diagnostic and therapeutic applications in this neurological subspecialty. Our institution receives weekly requests for advanced training in neuro-otology to prepare physicians for the societal demands of dealing with falls and other disorders of balance. Falls are a leading cause of mortality and fall prevention programs are being instituted globally. We have a long history of training physicians in neurological rehabilitation paradigms and have gathered world experts in these clinical areas that are qualified to give instruction in neuro-otology.

## Methods and Procedures

We identified the need to develop an e-learning system that allows us to deliver educational materials in both live virtual classrooms as well as an on-demand system. We needed a system that allowed us to implement a flipped learning classroom that complemented live learner participation in the virtual classroom. In order to do this, we needed to be able to have appropriate outcome measurements in the form of quizzes, tests, and interactive practical demonstrations. Our platform was designed to be appropriate to this pedagogical demand. We also needed a platform that was robust enough to allow us to screencast our classroom educational experience. We had to live stream our classes to a global audience that had different Internet speeds and different computers, tablets, and smart phones. Advances in technology have expanded the educational tools and learning options available to medical educators.

Jose Salazar noted that educators must be informed about the use of currently available educational technology tools to promote student retention, engagement, and interaction in online courses. He suggested that successful use of educational technology tools requires planning, organization, and use of effective learning strategies (17). We agree with this strategy and utilized a backwards design of our project. A backward design allowed us to create an educational content that allowed us to achieve our goals and promote the achievement of goals by our learners. The backward design starts with identification of the end learning goal or achievement and derives the curriculum from the skills necessary to achieve the learning goal. This is different from traditional curriculum development that begins with standardized texts that are not derived from the desired outcome. Once the educational content was chosen, we needed to choose the computer software that would best allow us to create and edit the screencast. Our intervention was the development of an e-learning platform that would deliver quality clinical training to neurologists located in various areas around the world. The e-learning platform was complemented with a flipped classroom paradigm.

We needed to use technology that allowed participants to access our learning content with a wide variety of their own personal technology. Our research of different methods of delivery identified that the Adobe Connect Learning systems (18) were ideal for our needs and encompassed all of the technology necessary to realize our project. The Adobe Connect platform allowed us to live stream our lecturers in a fashion that has a variety of windows or pods including live video and the ability to see the instructors as they speak. They also can view the instructor’s desktop or PowerPoint presentation at the same time and can involve themselves in interactive media. By utilizing combinations of a variety of pods, both the instructor and the learner could manipulate the learning environment to their best uses. We developed the ability to have individual learning rooms where we could group our learners into specific rooms with assignments and moderators. We provided our learners the ability to live stream while sharing their own video camera, microphone, and screens for a robust interactive experience. This technology was tried and tested and enabled us to implement our project without the necessity of developing our own interactive learning software.

Although the software contracts are expensive, we had the funding and the network to be able to implement this project. Our preliminary feedback from learners represents an excitement that is associated with great savings in time and expenses that ordinarily would be associated with travel to extension facilities throughout the world. Our review of the literature specific to the flipped classroom demonstrated that this pedagogical scenario was appropriate to maximize our efficiency and promote maximum learning.

## Interventions

An announcement of our neuro-otology program was made on our Institutional website and scholars were invited to apply for the program. All scholars had to be licensed healthcare professionals in active clinical practice. Four hundred seventy-three scholars (83 females and 390 males) applied to the program and 274 were randomized to this study. We block randomized the applicants into two main groups of 137 subjects with careful attention to include all of the female applicants equally in each group in order to provide a greater appreciation of any gender differences in our study. There are gender stereotypes that may affect training for physicians in medical specialties such as surgery. We wanted to know if there was a gender difference in neuro-otology training (19). We are also aware that male medical students tend to outperform females, while no gender differences were found among residents (20). We have a professional and societal need to measure any differences or bias in outcomes between males and females in neuro-otology training for non-neuro-otologists.

**Figure 1:**
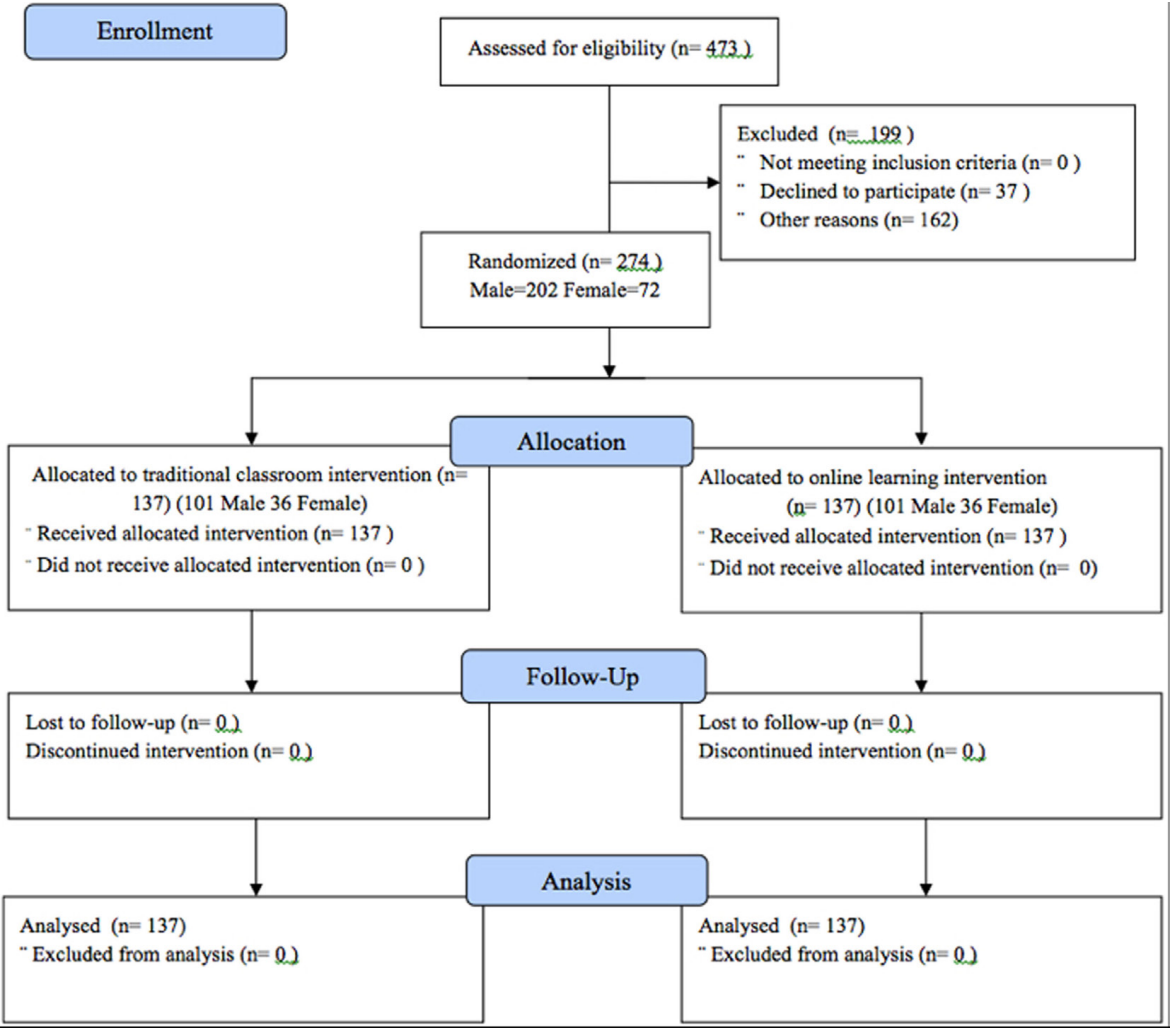
CONSORT study flow diagram.

Offers of acceptance into our program were made from our randomized total population. Thirty-seven subjects declined to participate, and 162 were not accepted due the sample and facility size limitations. Scholars that applied to the program and were not accepted were given the opportunity to attend the program at the next iteration (Figure 1).

All candidates took pre- and posttest multiple choice questions of the subject material at the beginning and end of course at the course location. Both pre- and posttests were multiple choice examinations of VOR, Movement, Head Turns, Head Tremor, Neurodegeneration, Inferior Olivary Complex, Collateral Projections, Eye Movement Training, Visual Saccades, Head Saccades, Visual Impairment, Walking Speed, Neuroprotection, Autophagy, Hyperkinetic Movement, Eye and Head Stability, Oscilllatory Head Movements, Gaze Stability, Leaky Neural Integrator, Cervical Dystonia, INC and Head Tilts, Visual Pursuits, Optokinetic Stimulation, and Vestibular Rehabilitation.

Our scholars gained exposure to educational material outside of the class and then used the class time to assimilate the knowledge through problem-solving exercises and discussions. They were able to hyperlink to their flipped classroom and the presentations and interaction were supported by a previous learning experience gained in the flipped classroom. Sessions were given in the classroom and in the online classroom synchronously using the Adobe Connect e-learning platform. Subjects randomized to the online classroom attended the sessions in a location of their choice and viewed the sessions on the Internet. The traditional classroom and synchronous online classroom did not review the course presentation or offer an on-demand learning paradigm. The educational experience was limited to their attendance in the two group classes. Two sample unpaired *t* tests with equal variances were calculated for all pretests and posttests for all groups and were controlled to include gender differences. Effect sizes were calculated using Cohen’s *d* analysis. The internal consistency of the study was estimated across repeated measures, and the sources of variance affecting the measurement were analyzed.

### Data Analysis and Statistics

We estimated sample sizes for a two-sample means test using our previous unpublished pilot study with a traditional classroom performance mean of 86.12, SD ± 5.01 and a controlled online mean performance of 84.20, SD ± 5.27. This revealed a Satterthwaite’s *t* test assuming unequal variances maintaining an alpha of 0.05 and a power of 80% suggesting a sample size of 274 with 137 subjects per group allowing for a 20% increase to address potential dropouts. Two sample unpaired *t* tests with equal variances were calculated for pretest and posttest results for all groups and were controlled to include gender differences. Effect sizes were calculated using Cohen’s *d* analysis. The internal consistency of the study was estimated across repeated measures, and the sources of variance affecting the measurement were analyzed. All analyses were performed using Stata/SE 14.0. For all tests, alpha (α) was set at 0.05.

### Ethics Statement

The study was approved by the Carrick Institute: Institutional Review Board HHS #: IRB00006615 FWA: 00022305 (CI #20170222001) and registered at Clinicaltrials.gov. ID:NCT03079349. Participants were not compensated and gave written informed consent before participation. There was equipoise.

## Results

There were 274 subjects included in this study with a mean age of 38.5 years with a minimum age of 24 and a maximum age of 64 representing 202 males and 72 females. Our subjects were composed of practicing physicians who desired to learn neuro-otological examination and treatment skills. These learners come from institutions that are globally represented by physicians from the United States, Canada, Australia, New Zealand, France, Spain, Holland, England, Norway, Denmark, Sweden, Spain, Portugal, Egypt, and Israel. A total of 473 scholars applied to the program.

We wanted to identify any differences in gender in medical education because of interest we have specific to gender bias in society. Of the applicants, 83 were female and 390 were male. We block randomized the applicants into 2 main groups of 137 subjects each with careful attention to include all of the female applicants in order to provide a greater appreciation of any gender differences in our study. In order to identify any gender bias, we randomized males and females into the two main groups that were further subdivided into four separate groups including two gender-specific traditional classrooms as a control and two gender-specific synchronous online learning experience as an active treatment group. Offers of acceptance into our program into both main groups were made from our randomized total population until we had the necessary numbers of subjects that accepted a position to conduct our study. Scholars that applied to the program but were not accepted into groups were given the opportunity to attend the program at another time or online in a non-synchronous fashion.

The accepted subjects were enrolled in a neuro-otology clinical program of education of 25 h held over a 3-day period. The educational settings for our course were varied due to individual scholar location. Our program included hands on practical experience presented in the clinical setting with patients, patient scenarios, and patient simulators. The live synchronous streaming learners needed to have patients or simulated patients or colleagues to simulate the case. Potential problems of time zones and dates, including holidays, were taken into consideration with start times of 0800 EST working well for all learners throughout the world. The Europeans were able to meet in the afternoon or early evening, while North Americans were able to meet during the morning time. Participants in Australasia were in a time zone that is roughly 13–14 h ahead of the Eastern Standard Time of our institution. Their time zone is approximately 6 h ahead of the European time zones. The early morning American participation, coupled with the afternoon European anticipation and the late evening Australasian participation worked well for our learners.

An analysis of pre- and post-course examinations was conducted and included the differences between pre- and posttest scores for all groups. The groups included global test scores without delineation of gender, composed of a control (live classroom scores) and a treatment (online streaming scores), gender-specific scores composed of a control (live classroom scores), and a treatment (online streaming scores). There was strong statistical significance in outcomes of the pre-and post examination scores for all groups of learners with *p* < 0.001. The effect sizes were moderate for all groups with Cohen’s *d* of −4.45 to 5.38 (Table 1).

**Table 1:** Pre- and posttest scores and the significance of the difference between groups.

Type of score	Observations	Mean ± SD	Minimum–maximum	*P*-value	Cohen’s *d*
**Comparison of test scores in all subjects**					
– Overall pretest – Overall posttest	274 274	47.6 ± 10.1 86 ± 5.1	15–80 70–99	**<0.001**	−4.78

Live classroom scores					
– Pretest – Posttest	137 137	46.9 ± 9.8 86.1 ± 5	15–75 75–99	**<0.001**	−5.02

Online classroom scores					
– Pretest – Posttest	137 137	48.3 ± 10.4 86 ± 5.3	32–80 70–98	**<0.001**	−4.56

**Comparison of test scores in male subjects**					
– Overall pretest – Overall posttest	202 202	47.7 ± 10.5 86 ± 7	15–80 71–98	**<0.001**	−4.30
– Live classroom pretest – Live classroom posttest	101 101	46.9 ± 46.9 85.6 ± 85.6	15–75 75–99	**<0.001**	−4.90
– Online classroom pretest – Online classroom posttest	101 101	48.5 ± 48.5 86.4 ± 86.4	32–80 70–96	**<0.001**	−4.59

**Comparison of test scores in female subjects**					
– Overall pretest – Overall posttest	72 72	47.4 ± 47.4 86.4 ± 86.4	30–70 71–98	**<0.001**	−4.88
– Live classroom pretest – Live classroom posttest	36 36	46.9 ± 46.9 87.6 ± 87.6	30–70 75–98	**<0.001**	−5.38
– Online classroom pretest – Online classroom posttest	36 36	47.8 ± 47.8 85.1 ± 85.1	36–70 15–98	**<0.001**	−4.59

*All subjects demonstrated learning by examination with statistical significance bold*.

**Figure 2:**
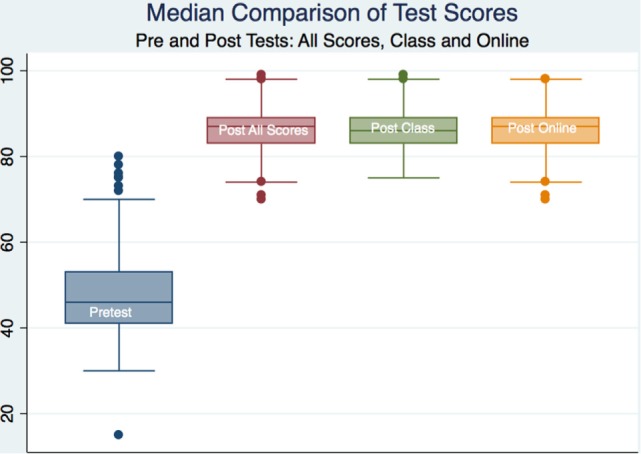
Box plots of pre- and post-scores for all subjects.

**Figure 3:**
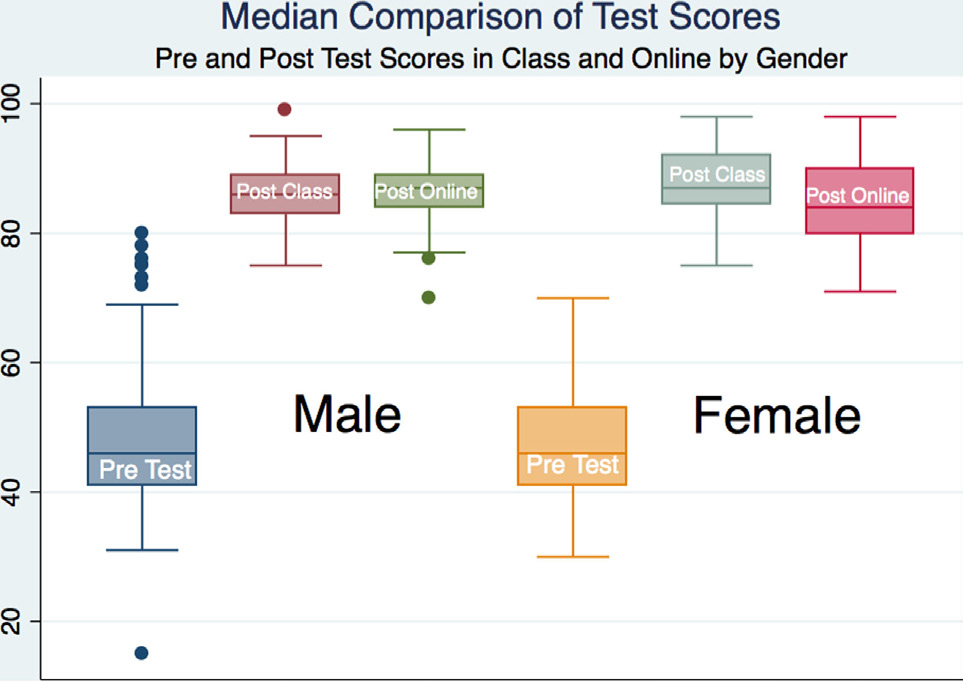
Box plots of pre- and post-scores for all subjects and by gender.

All subjects demonstrated learning by examination with statistical significance; however, there was no statistical or substantive significance between groups save for a statistical difference in total outcome scores between males and females that attended the traditional classroom (*p* < 0.05) with a moderate effect size (Cohen’s *d* = −0.407). The female learners in the traditional classroom scored higher than the male learners. We looked at the total scores on the pre- and posttests and did not compare outcomes on individual questions or categories (Table 2; Figures 2 and 3).

**Table 2:** Pre- and posttest scores: two-sample *t* tests with equal variances, unpaired.

Type of score	Obs	Mean	SE	SD	*t*	DOF	95% CI	*P*-value	Cohen’s *d*
Post live score	137	86.12	0.4282	5.01	0.1763	272	85.27722–86.97096	0.8602	0.021
Post online score	137	86.01	0.4497	5.26			85.12529–86.90391		
Post total score	274	86.06	0.3099	5.13	0.1011	409	85.45917–86.67952	0.9195	0.007
Post online score	137	86.02	0.4497	5.26			85.12529–86.90391		
Post total score	274	86.07	0.3099	5.13	−0.1028	409	85.45917–86.67952	0.9182	−0.010
Post live score	137	86.01	5.26	70			85.27722–86.97096		
Pre total male	202	47.71	0.7356	10.46	0.2555	272	46.26724–49.16840	0.7985	−4.30
Post total female	72	85.97	7.00	71			45.17077–49.55145		
Post live male	101	85.59	0.4722	4.75	−2.0989	135	84.65716–86.53096	**0.0377**	−0.407
Post live female	72	86.35	6.38	71			85.75211–89.47011		
Post online male	101	86.35	0.4460	4.48	1.2388	135	85.46172–87.23135	0.2176	−4.90
Post online female	101	85.59	4.75	75			82.71367–87.45300		

*Test scores: two sample t tests with equal variances unpaired statistical significance bold*.

## Discussion

This investigation involves the design of a novel synchronous educational program utilizing current technology that was compared to traditional classroom teaching. Our instructional program was designed to address neuro-otology education in the classroom and synchronously at a distance while providing the evidence-based methodology to evaluate both mechanisms of learning. There was no statistically significant difference in outcomes between our synchronous online training and the traditional classroom experience. Our contribution to the understanding of outcomes associated with classroom and synchronous online learning may benefit society in general as well as all professional stakeholders. Our objective was to determine if learning in a synchronous online classroom would result in similar outcomes as the traditional classroom. Our hypothesis that learning in an online classroom utilizing best educational practices that are dependent upon best technology would be equal to learning in a traditional classroom was supported by our data.

There are current concerns that learning in an online environment might not represent the quality of education received in a traditional classroom. Our study answers this question and promotes a confidence in learners that there is no difference in outcomes between training in the traditional classroom and online synchronous education. The flipped classroom complemented our desire to improve the learning environment. Our scholars gained exposure to new material outside of the class and then used the class time to assimilate the knowledge through problem-solving exercises or discussion that was robust as a consequence of our technology. They were able to hyperlink to their flipped classroom and the presentations and interaction were supported by a previous learning experience gained in the flipped classroom. The understanding of material before the course sessions promoted an increased quality of understanding of the subject with increased quality of participation and engagement in discussions.

Educational solutions increasingly need to be timely, efficient, pragmatic, high quality, and aligned to the needs of the professional in a specific context, sustainable, and cost-effective (21). Katherine Barsness and colleagues identified that medical education must address all potential cognitive and technical performance gaps, professionalism, and personal behaviors, as well as effective team communication (22). They suggested that educational strategies should seek to replicate the stressors and distractions that might occur during a high-risk operation or critical care event. They criticized the traditional medical educational model of “See one, do one, teach one,” noting that patients are exposed to the risk of harm inherent to any learning curve. Barsness suggests that the majority of educational goals can be achieved with the addition of simulation-based education as a valuable adjunct to traditional training methods. Our instructional intervention incorporated instructional technology tools in a meaningful way by using a blended approach to education that included Merrill’s first principles of instruction. Our instructional methodology was problem-based and involved the student in distinct phases of learning. Similar to the Kolb cycle of learning, we started our class presentation by activating prior experiences of the learner. We accomplished this with a flipped classroom methodology that allowed the learner to prepare himself/herself for the practical/clinical and didactic portions of the program. We expected that the learner is able to identify strengths and weaknesses that would promote long-term learning as a consequence of the activation of their prior experiences. We introduced the “what” of the material following the first section of instruction. This knowledge base was referred to as the breath and death of instruction that was necessary to translate the two applications.

Medical education should address the different cultures of learning of the medical student when they prepare their instructional methods and teaching materials to fulfill the educational needs of culturally diverse students (23). We recognized the importance of this with a global diverse group of scholars attending our synchronous online sessions. Our educational program included a variety of methods and tools to enhance learning of a considerable amount of complex material and to promote critical thinking and decision making that is not promoted by traditional didactic teaching methods that remain as the predominant teaching strategies in health-care education (24). The increasing availability of newer technology in higher education such as video streaming and podcasting provided us the opportunity to utilize a variety of approaches to cater for a wider range of learning styles. Students find hybrid learning strategies that combine traditional teaching with more innovative methods particularly beneficial (25).

The rapid advancement of computer and information technology in recent years has resulted in the rise of e-learning technologies to enhance and complement traditional classroom teaching in many fields (26). Hugh Silk and colleagues at the University of Connecticut Health Center noted that medical educators need to teach learners to efficiently access the best available evidence at the point of care and apply it in a patient-centered manner. They realized that as information becomes more readily available *via* the Internet and handheld computers, strategies to use these tools as part of the educational process become more important. They concluded that new teaching skills are needed when attempting to seamlessly introduce technology in the midst of blending old and new teaching methods (27). Our synchronous online neuro-otology program can be viewed on smart phones, tablets, and computers in any location that has an Internet signal. The teaching skills developed in this course included an interactive pedagogy that kept the learner engaged by requiring participation.

We promoted feedback from our learners with the construction of interactive tests that were designed for the specific criterion involved in the neurological physical examination. These tests served as our summative evaluation, whereas the formative evaluation was continuous by its presence in all parts of the instructional systems design. We utilized standard measures of competency by instituting pre- and post-learning examinations and demonstrations. Our investigation promoted the use of a mixed methodology of inquiry. Our competency examinations lead to well-established quantitative methodology has allowed us to make statements specific to statistical and substantive significance in our study. We also desired to understand the common experiences of learners in a group in order to further develop our policies and practices that will affect the delivery of our pedagogy. A phenomenological qualitative approach has allowed us to accomplish these goals and will be published in a follow-up article.

We have identified that women score higher than men in a traditional classroom environment studying neuro-otology but score similarly in the synchronous online classroom. Other investigators have found gender differences in outcomes of medical training. For example, clinal clerkship grades in a 6-week obstetrics and gynecology have been shown to be significantly higher for females than for males and males scored lower on OSCE, oral, and NBME subject examinations (28). The difference in final grades between genders necessitates further investigation. We desire to study this phenomenon and suggest that the differences in traditional classroom performance might be associated with the physical interaction between men and women that does not occur online. Gender-specific variables will be explored using a mixed methodology of inquiry including both ethnographic and phenomenological approaches in our continued investigation of this phenomenon.

## Strengths and Limitations

This study represents a successful outcome of training in neuro-otology that might not be representative of other medical education. The subjects in the study were all experienced clinicians with prior training in neurology. The outcomes of this study might be different with a different cohort of health-care providers. There are several advantages or strengths to both learners and faculty involved in our synchronous online learning, for example. The ability to attend a clinical program without the need of travel and in a location of personal choice appears to increase access to the learning program. Physicians that would not be able to attend the onsite classroom would not have the opportunity to benefit from the learning experience without this opportunity. Thus, we feel that the quality of learning and the opportunity to develop a self-directed and lifelong learning experience is enhanced with the ease of attending educational sessions in an online environment. The advances in the software enabling our program are associated with a facilitation of information exchange in a fashion not typical in a traditional classroom. Hyperlinks to articles, videos, and demonstrations are robust as is the ability to communicate in real time with other learners in private chats without disturbing colleagues as in a traditional classroom. The ability to post questions and have faculty responses in real time are robust as is the experience of breakout rooms and group learning experiences.

There are several limitations that we have noted with the development of our online synchronous program. The startup cost of software, hardware, and technical training for our institution was expensive and perhaps prohibitive to institutions that are not well funded. There is a demand of time for faulty training and preparation that is greater than that customarily embraced in the traditional classroom. Faculty must prepare themselves for the technical demands of live synchronous streaming of their presentations and coordinate interactions with learners in addition to the demands of presentation of the subject material. The utilization of interactive polling and breakout rooms demands a fluid technical ability of the faculty and support staff, something that is not necessary in the traditional classroom. We found that there are always technical issues of our learners in the program that need to be addressed in a quick and effective manner. Problems with Internet speeds and manipulation of the software communication tools including learner cameras and microphones demand a talented and qualified support staff that might problem solve in real time. We had extensive searches of companies and personnel before we were able to build a quality team. We also needed to run many simulations that would involve attending to technical problem with backup procedures and equipment.

## Conclusion

There is a need to increase learning and to embrace the change in pedagogical methodology in medical education. Traditional classroom learning has not changed much in the last 100 years in spite of individual studies that show promise for various components of educational reform. This study combined the most current methodology in medical education with novel delivery technological services. The value of the qualitative portion of this investigation has guided us toward instituting change necessary for future studies and implementation of the consequences of this investigation. There has been an explosion of online learning available from most institutions of higher learning in the United States and globally. There has not been good quantitative and qualitative evaluation of these courses.

Online learning is a currently technically available pedagogy that has been demonstrated to equal the outcomes obtained in the traditional classroom. Physicians might choose to attend an online synchronous learning experience that has been associated with good outcomes of effective earning. Our delivery of online synchronous learning scenarios has been demonstrated to be as effective as competent traditional classroom learning. There is scant literature specific to this exploding phenomenon of Internet delivery of education. The contribution of this study to an evidence-based model of medical education promotes the embrace of a novel pedagogy that ultimately may improve physician services to humankind by providing easier access to clinical education. We can expect to see a change in learning strategies with cost savings to individuals and society specific to medical education. These changes may be associated with a decreased need for brick and mortar facilities as well as travel and expense of living for students. We feel that there is a danger of implementation of novel technology without the benefit and experience of good qualified evaluation of the consequence of that intervention. We are hopeful that this model might contribute to the science of medical education and the training of physicians through improvement in pedagogical models and educational delivery.

The literature is scant specific to outcome measurements of medical education given at a distance in a synchronous fashion. We hope that this study might encourage other medical educators to test their outcomes so that physicians might be able to choose online courses with greater confidence in their educational and clinical integrity.

## Author Contributions

The authors contributed in the design, analysis and interpretation of data, and review and drafting of the article.

## Conflict of Interest Statement

The authors declare that the research was conducted in the absence of any commercial or financial relationships that could be construed as a potential conflict of interest.
